# Treatment preferences of adults and adolescents with alopecia areata: A discrete choice experiment

**DOI:** 10.1111/1346-8138.17056

**Published:** 2023-12-12

**Authors:** Tommi Tervonen, Chiara Whichello, Ernest Law, Jonathan Mauer, Debanjali Mitra, Myrto Trapali, Nicolas Krucien, Brett Hauber

**Affiliations:** ^1^ Kielo Research Zug Switzerland; ^2^ Evidera Inc. London UK; ^3^ Pfizer Inc. New York City New York USA

**Keywords:** alopecia areata, discrete choice experiment, janus kinase inhibition

## Abstract

PRODUCTS with janus kinase (JAK) inhibition have been shown to promote hair regrowth in patients with alopecia areata (AA). To guide drug‐approval and treatment decisions, it is important to understand patients' willingness to accept the potential risks of JAK inhibition in exchange for potential benefits. We quantified the treatment preferences of adult (≥18 years) and adolescent patients (12–17 years) with AA in the US and Europe to determine the trade‐offs they are willing to make between benefits and risks. Preferences for oral AA treatment attributes were elicited using a discrete choice experiment consisting of 12 tasks in which patients chose between two hypothetical treatment alternatives and no treatment. Benefits included the probability of 80%–100% scalp hair regrowth (Severity of Alopecia Tool score ≤ 20) and achieving moderate‐to‐normal eyebrow and eyelash hair. Treatment‐related risks included 3‐year probabilities of serious infection, cancer, and blood clots. Preference estimates were used to calculate the maximum level of each risk that patients were willing to accept for increases in treatment benefits. The most important attribute to both adults (*n* = 201) and adolescents (*n* = 120) was a 50% probability of achieving hair regrowth on most or all the scalp; however, adolescents placed greater relative importance on this attribute than did adults. Adults were averse to the risks of serious infection, cancer, and blood clots, whereas adolescents were averse to the risk of cancer. For a 20% increase in the probability of 80%–100% scalp hair regrowth, adults were willing to accept a mean (95% confidence interval) 3‐year risk of serious infection, cancer, and blood clots of 7.4% (5.5–9.3), 2.5% (1.9–3.1), and 9.3% (6.4–12.2). Adolescents were willing to accept a 3‐year risk of cancer of 3.3% (2.4–4.2). Patients with AA in the US and Europe are willing to accept substantial risks to obtain an effective treatment.

## 1 INTRODUCTION

Alopecia areata (AA) is an autoimmune disease that causes hair loss. AA can affect up to 2% of the population in their lifetime and can affect males and females of any age.^1^ AA can be limited to round or oval patches of hair loss or cause loss of all scalp hair (alopecia totalis) or all body hair (alopecia universalis).^2^ Patients with AA suffer from stigmatization because of the poor cultural perception of hair loss, resulting in social, economic, and emotional burdens.^3^ Approximately half of the patients with AA report reduced quality of life, and nearly 70% experience psychiatric disorders, especially depression, anxiety, and even suicidal ideation.^1^, ^3^ Patients with extensive AA frequently experience adverse psychological effects and psychosocial distress.^1^

Until recently, AA treatments have had limited success and no cure has been found.^1^ In the last decade, several case reports and small studies reported hair regrowth in patients treated with different systemic or topical therapies with Janus kinase (JAK) inhibition.^4^ The ALLEGRO phase 2b/3 trial (NCT03732807) results demonstrate that ritlecitinib, an oral inhibitor of JAK3/tyrosine kinase expressed in hepatocellular carcinoma (TEC) kinase family, promotes hair regrowth in patients with AA.^5^, ^6^ The BRAVE‐AA1 and BRAVE‐AA2 trials show that baricitinib is superior to placebo at promoting hair regrowth.^7^ However, approved and marketed JAK inhibitors have known risks (e.g., serious infections, malignancies, and thromboembolic events) and their long‐term safety is not fully known.^4^

The emergence of JAK inhibition therapies is an important step forward in the treatment of AA. However, the willingness of patients to accept the risks associated with JAK inhibition in exchange for potential treatment benefits has not been evaluated. Such preference information is increasingly used to guide the development of new treatments, inform clinical guidelines and regulatory decisions, supplement health technology assessments, and facilitate shared decision‐making at the point of care.^8^, ^9^, ^10^ In a 2018 report based on perspectives of adult and pediatric patients with AA, the US Food and Drug Administration (FDA) emphasized that patient perspectives should be considered in the development of new AA treatments and guide treatment management.^11^ Patient preferences for different treatment attributes can be quantified using a discrete choice experiment (DCE) in which patients are asked to choose between two or more hypothetical treatment options in each of a series of choice questions.^12^, ^13^ Here, we describe the results of a DCE administered to patients with AA in the US and five European countries to quantify their preferences for treatment benefits and risks and the trade‐offs they are willing to make between them.

## 2 METHODS

### 2.1 Study design

Instrument development, data collection, and analysis followed the good practice guidelines published by the International Society for Pharmacoeconomics and Outcomes Research and the FDA.^14^, ^15^, ^16^, ^17^, ^18^ A DCE was used to elicit the preferences of patients with AA for the benefit and risk attributes associated with JAK inhibition. This study adopted a mixed‐method approach that combined qualitative and quantitative research methods to design the DCE based on a detailed targeted literature review, in‐depth qualitative patient interviews, and consultation with clinical experts. Attribute levels reflecting AA treatments were informed by a review of the characteristics of marketed medicines with JAK inhibition (none of which was approved for the treatment of AA at the time this study was conducted) and data from phase 1 and 2 clinical trials of ritlecitinib. Cognitive pretest interviews and a quantitative interim analysis were conducted to ensure that the DCE was performing as intended. The FDA was formally consulted through a Type C meeting process to ensure that the concerns of regulators were addressed during the study development and execution (Data S1).^19^

The study included adult (aged ≥18 years) and adolescent (aged 12–17 years) patients with AA. Adolescents aged 12–14 years completed the survey with the help of a caregiver. Patients who participated in the study were not recruited based on their participation in any clinical trial. Study invitations were sent to physician networks and local study recruiters to refer participants. Interested participants answered an online prescreening questionnaire to determine their eligibility.

Patients had to have a dermatologist‐confirmed diagnosis of alopecia totalis, alopecia universalis, or ≥50% scalp hair loss due to AA including alopecia totalis and alopecia universalis; be aged ≥12 years; be a resident of the US, UK, France, Germany, Italy, or Spain; be able to read, speak, and write in the local language of the relevant country; and provide online consent to participate in the study. Patients could not have any of the following: a cognitive impairment or acute psychopathology that could interfere with their ability to provide consent; types of alopecia or causes of hair loss other than AA as diagnosed by a dermatologist; or other diseases that affect the scalp or skin on the scalp as diagnosed by a healthcare professional. The study was approved by Ethical and Independent Review Services on October 17, 2019, for the qualitative phase (ID: 19151‐01) and July 17, 2020 for quantitative phase (IDs: 20108‐01 and 21130‐01).

### 2.2 Survey content

The DCE was presented as part of an online survey. Patients were presented with a description of the overall choice setting as a hypothetical scenario, an introduction to each treatment attribute, and a question to aid in understanding and interpreting probability data. Patients were then presented with the DCE, followed by questions about health literacy and numeracy, their experience with AA, treatment for AA, overall health, and sociodemographic characteristics (Data S1).^20^, ^21^ All patients also completed the Hospital Anxiety and Depression Scale (HADS), the 3‐items version of Patient Satisfaction with Hair Growth (P‐SAT) scale, and the Alopecia Areata Patient Priority Outcomes (AAPPO) scale (Table S1).^22^

### 2.3 DCE design

The set of attributes and levels included in the DCE are shown in Table 1. The levels of each attribute were informed by a review of the characteristics of marketed JAK inhibitors (none of which was approved for the treatment of AA at the time this study was conducted),^4^, ^23^, ^24^ as well as data from phase 1 and 2 clinical trials of ritlecitinib. The attribute levels were tested for comprehension and appropriateness during the cognitive pilot interviews and quantitative pilot testing. Patients were required to complete a series of choice tasks, each of which consisted of three alternatives: two unlabeled hypothetical treatment alternatives described by combinations of attribute levels (Treatment A and Treatment B) and a no‐treatment option.^25^, ^26^ The no‐treatment option was fixed and was defined by 0% probability of hair regrowth and 0.1% 3‐year probability of each risk. The lowest risk level of 0.1% was intended to capture the non‐zero baseline/background level of risk in the no‐treatment option. When the no‐treatment option was chosen, a follow‐up question was presented, asking patients to choose between Treatment A and Treatment B from the previous question.

**TABLE 1:** Attributes and levels.

Attribute	Description	Levels
Hair on most or all your scalp	The chance of getting most or all your scalp hair (80%–100% of your scalp hair) after 24 weeks on treatment	0% (0 out of 1000 patients)
		10% (100 out of 1000 patients)
		30% (300 out of 1000 patients)
		50% (500 out of 1000 patients)
Eyebrows	The chance of getting moderate (mildly decreased density and/or short gaps in the eyebrows) or normal eyebrows after 24 weeks on treatment	0% (0 out of 1000 patients)
		20% (200 out of 1000 patients)
		40% (400 out of 1000 patients)
Eyelashes	The chance of getting moderate (mildly decreased density and/or short gaps in the eyelashes) or normal eyelashes after 24 weeks on treatment	0% (0 out of 1000 patients)
		20% (200 out of 1000 patients)
		40% (400 out of 1000 patients)
Risk of serious infections during 3 years of treatment	A serious infection means that you may have to stay in hospital for treatment of the infection and/or receive treatment through an injection. The serious infection may potentially be life‐threatening. You may need to temporarily (until the infection has cleared) or permanently stop your treatment for alopecia areata. Examples of such infections may include lung infection, shingles, urinary tract infection etc.	0.1% (1 out of 1000 patients treated for 3 years)
		3% (30 out of 1000 patients treated for 3 years)
		6% (60 out of 1000 patients treated for 3 years)
Risk of cancer during 3 years of treatment	Cancer typically requires chemotherapy or surgery, and some cancers can be life‐threatening. Some cancers can be treated or cured with treatment while others may not be treatable. You may need to temporarily or permanently stop your treatment for alopecia areata	0.1% (1 out of 1000 patients treated for 3 years)
		0.5% (5 out of 1000 patients treated for 3 years)
		2% (20 out of 1000 patients treated for 3 years)
Risk of blood clots during 3 years of treatment	Blood clots require treatment with blood thinning medication, may require you to stay in hospital for treatment, and in some cases may potentially be life‐threatening. You may need to temporarily or permanently stop your treatment for alopecia areata	0.1% (1 out of 1000 patients treated for 3 years)
		2% (20 out of 1000 patients treated for 3 years)
		6% (60 out of 1000 patients treated for 3 years)

**FIGURE 1:**
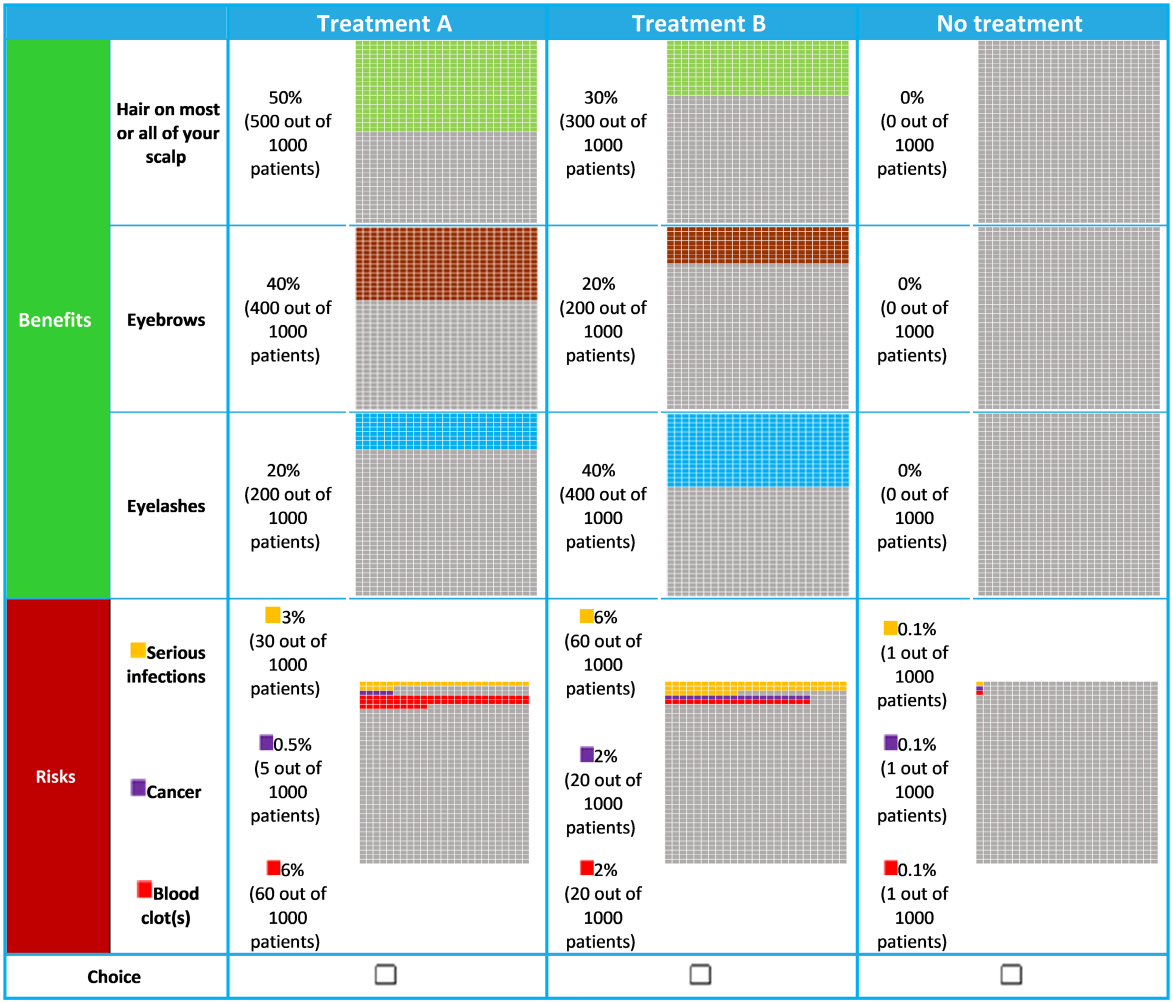
Example of a discrete choice experiment question.

Attributes were grouped into “benefits” and “risks”. Across patients, the benefits were randomized to appear either before or after the risks.^27^ The three benefit attributes always appeared in the same order, as did the three risk attributes. An example of the choice task is shown in Figure 1.

A D‐efficient design including 24 experimental tasks was generated in Ngene version 1.2.1 (choice‐metrics.com). The experimental tasks were split into two blocks of 12 choice tasks. Patients were randomly allocated across blocks, and the order of the experimental tasks was randomized between patients.

Each patient also completed two non‐experimental tasks assessing choice consistency (Task 13 was a repetition of Task 1) and engagement in the survey (Task 14 was a dominated choice task, where a high‐benefit, low‐risk treatment was compared to a low‐benefit, high‐risk treatment; a participant was said to fail the dominance test when they chose the inferior or dominated option as the preferred treatment).^28^, ^29^ Model estimates were used to predict probability of failing or passing the test as described previously.^30^ DCEs require participants to make trade‐offs between the attributes by adopting compensatory decision‐making (i.e., a deterioration in one attribute can be compensated by improvement in another attribute). This is typically not the case when participants make decisions dominated by a single attribute or always select the same option. A participant was deemed to have a dominant preference for a particular attribute if they always chose an alternative with a higher or lower level of that attribute, regardless of the levels of the other attributes.

### 2.4 Statistical analysis

Discrete choice experiment data were analyzed within the random utility maximization framework with an interacted error component multinomial logit model.^29^, ^31^, ^32^ In this model, the treatment preferences of adult and adolescent patients were jointly analyzed. The model primarily measured average sensitivities to marginal changes in the treatment attributes (e.g., effect of increasing probability of scalp hair regrowth by 1%). Interaction effects between the attributes' levels and the type of patients (i.e., adult vs. adolescent) were included in the model to allow for differences in sensitivities between adult and adolescent patients. The individual error component was added to account for the panel nature of the choice data. Only the initial choices among the three options were used for the modeling of treatment preferences.

The estimated sensitivities to attribute changes were then used to derive scores of relative attribute importance (RAI) and measures of maximum acceptable risk (MAR). The RAI scores measured the importance of an attribute relative to all other attributes conditional on the range of levels included in the study. The Krinsky‐Robb procedure in 10 000 iterations was used to compute 95% confidence intervals (CIs) for RAI scores.^33^ MAR quantified the trade‐offs that patients were willing to make between the benefit attributes and each risk attribute. The Delta method was used to obtain the 95% CIs (Data S1).^34^

## 3 RESULTS

### 3.1 Patients

The study included 201 adults (median age 39 years) and 120 adolescents (median age  15 years) (Table 2). Most adults (94%) and adolescents (89%) reported extensive scalp hair loss, scoring ≥2 on the AAPPO scalp hair loss item in which 0 indicates “no hair loss” and 4 indicates “complete hair loss”. The average AAPPO emotional symptom score was significantly higher for adolescents (2.69) than for adults (1.93; *p* < 0.001), indicating adolescents reported a greater impact of AA on their emotional symptoms and activity limitations. More than one half of patients were currently using an approved treatment for alopecia areata. Ninety‐six percent of adults and 89% of adolescents had adequate numeracy (scores ≥3), and 85% of adults had adequate health literacy (scores >2) (adolescents were not tested for health literacy).

**TABLE 2:** Sample characteristics.

	Adults				Adolescents			
	Overall (*N* = 201)	Europe (*N* = 139; 69%)	US (*N* = 62; 31%)	*p* value	Overall (*N* = 120)	Europe (*N* = 59; 49%)	US (*N* = 61; 51%)	*p*‐value
Age, median (interquartile range)	39 (29–52)	43 (31–53)	35 (26–45)	0.001	15 (14–16)	15 (13–16)	15 (14–16)	0.457
Male, *n* (%)	71 (35)	59 (42)	12 (19)	0.003	60 (50)	31 (53)	29 (48)	0.715
Education level, *n* (%)				0.001				
Less than high school	5 (2)	5 (4)	0 (0)		–	–	–	–
High school	68 (34)	60 (43)	8 (13)		–	–	–	–
Some college/university	29 (14)	10 (7)	19 (31)		–	–	–	–
College/university/postgraduate degree, *n* (%)	99 (49)	64 (46)	35 (56)		–	–	–	–
Time since AA diagnosis, *n* (%)				0.001				0.001
<1 year	19 (9)	16 (11)	3 (5)		25 (21)	24 (41)	1 (2)	
1–5 years	71 (35)	53 (38)	18 (29)		48 (40)	23 (39)	25 (41)	
>5 years	111 (55)	70 (0)	41 (66)		47 (39)	12 (20)	35 (57)	
Area of hair loss, *n* (%)								
Eyebrows	134 (67)	84 (60)	50 (81)	0.008	50 (42)	30 (51)	20 (33)	0.069
Eyelashes	121 (60)	77 (55)	44 (71)	0.054	25 (21)	11 (19)	14 (23)	0.722
Rest of the face	88 (44)	55 (40)	33 (53)	0.099	16 (13)	7 (12)	9 (15)	0.844
Rest of the body	112 (56)	66 (47)	46 (74)	0.001	17 (14)	9 (15)	8 (13)	0.941
AAPPO score								
Emotional symptoms^a^, mean (SD)	1.93 (1.28)	1.89 (1.29)	2.00 (1.28)	0.562	2.69 (1.20)	2.88 (1.09)	2.51 (1.28)	0.095
Activity limitations^b^, mean (SD)	0.76 (1.02)	0.84 (1.08)	0.56 (0.85)	0.046	1.51 (1.22)	1.74 (1.01)	1.28 (1.35)	0.036
Moderate to complete hair loss^c^, *n* (%)								
Scalp	188 (94)	127 (91)	61 (98)	0.066	107 (89)	54 (92)	53 (87)	0.600
Eyebrows	135 (67)	83 (60)	52 (84)	0.001	30 (25)	15 (25)	15 (25)	0.999
Eyelashes	118 (59)	73 (53)	45 (73)	0.012	23 (19)	9 (15)	14 (23)	0.402
Current use of approved treatments for AA, *n* (%)								
Betamethasone topical	9 (4)	8 (6)	1 (2)	0.276	–	–	–	NC
Clobetasol topical	25 (12)	23 (17)	2 (3)	0.016	–	–	–	NC
Desoximetasone topical	5 (2)	3 (2)	2 (3)	0.999	–	–	–	NC
Triamcinolone injection	6 (3)	5 (4)	1 (2)	0.663	–	–	–	NC
Hydrocortisone injection	2 (1)	2 (1)	0 (0)	0.573	–	–	–	NC
Prednisolone	7 (3)	6 (4)	1 (2)	0.444	–	–	–	NC
Prednisolone injection	4 (2)	3 (2)	1 (2)	0.999	–	–	–	NC
Methylprednisolone	5 (2)	4 (3)	1 (2)	0.685	–	–	–	NC
Methylprednisolone infusion	2 (1)	2 (1)	0 (0)	0.574	–	–	–	NC
Dexamethasone	5 (2)	4 (3)	1 (2)	0.683	–	–	–	NC
Squaric acid dibutylester	8 (4)	7 (5)	1 (2)	0.435	–	–	–	NC
Diphenylcyclopropenone	3 (1)	3 (2)	0 (0)	0.551	–	–	–	NC
Anthralin/Dithranol	1 (0)	1 (1)	0 (0)	0.999	–	–	–	NC
Minoxidil	14 (7)	11 (8)	3 (5)	0.561	–	–	–	NC
Cyclosporine	4 (2)	4 (3)	0 (0)	0.312	–	–	–	NC
Sulfasalazine	1 (0)	1 (1)	0 (0)	0.999	–	–	–	NC
Methotrexate	6 (3)	6 (4)	0 (0)	0.18	–	–	–	NC
Prednisolone	7 (3)	6 (4)	1 (2)	0.444	–	–	–	NC
Tablets or pills^d^	0 (0)	–	–	NC	68 (57)	35 (59)	33 (54)	0.694
Cream on your skin^d^	0 (0)	–	–	NC	69 (58)	40 (68)	29 (48)	0.039
Injections^d^	0 (0)	–	–	NC	11 (9)	3 (5)	8 (13)	0.227
Other	24 (12)	16 (12)	8 (13)	0.964	15 (12)	2 (3)	13 (21)	0.007
None	104 (52)	55 (40)	49 (79)	0.001	16 (13)	8 (14)	8 (13)	0.999

Abbreviations: AA, alopecia areata; AAPPO, Alopecia Areata Patient Priority Outcomes; NC, not calculated; SD, standard deviation; US, United States.

^a^ Patients rated the impact of AA over the previous week on a 5‐point scale from 0 (“never”) to 4 (“always”).

^b^ Patients rated their experiences over the previous week on a 5‐point scale from 0 (“not at all”) to 4 (“completely”).

^c^ Patients rated their hair loss on a scale from 0 (“no hair loss”) to 4 (“complete”).

^d^ Treatment options were presented to adolescents as type of treatment because cognitive pilot interviews demonstrated that adolescents found it difficult to identify or recall the names of the specific AA treatments they were currently using.

### 3.2 Internal validity

Most patients (94% of adults, 66% of adolescents) did not have dominant attribute preferences, indicating that patients made trade‐offs across multiple attributes in their treatment choices. Six percent of adults and 32% of adolescents made choices predominantly based on the attribute “hair on most or all of your scalp” (Table S2).

Eighty‐nine percent of adults and 96% of adolescents passed the dominated choice test. Both were higher than expected probabilities of passing the test (72% and 88%, respectively), indicating that patients were likely to have had a good understanding of attributes and their levels.^30^ Over three‐quarters of adults (78%) and adolescents (78%) passed the stability test (i.e., made the same choices to the questions when repeated) with observed failure rates comparable to other health DCEs in the literature.^35^ Only two adults and two adolescents always selected Treatment A or Treatment B, and 16 adults (8%) and 1 (1%) adolescent always chose “No treatment.”

### 3.3 Patient preferences for treatment attributes

**FIGURE 2:**
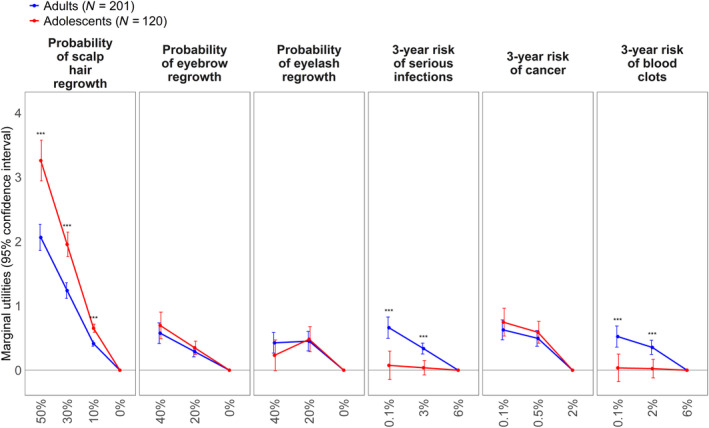
Average sensitivities to marginal changes in the treatment attribute levels.

Increases in the probabilities of scalp hair regrowth and eyebrow regrowth and decreases in the probabilities of all risks were positively evaluated by patients (Figure 2 and Table S3). For adolescents, increases in the probabilities of scalp hair regrowth and eyebrow regrowth and decreases in the probability of cancer had positive preference weights. However, decreases in the 3‐year risks of blood clots and serious infections did not result in higher preference weights for adolescents. Risks of serious infection and blood clots were not important to adolescent patients. The two probabilities of eyelash regrowth (20% and 40%) were disordered (i.e., the preference weight for a 20% probability of eyelash regrowth was higher than that of a 40% probability of eyelash regrowth; however, the difference was not statistically significant [*p* < 0.1]).

The most important attribute for both adults and adolescents was the probability of scalp regrowth after 24 weeks of treatment from 0% to 50% (adult RAI 42.1% [95% CI, 38.7–45.3]; adolescent RAI  61.6% [95% CI 56.2–65.1]) (Table S4). For adult patients, this was followed by reducing the risk of serious infection during 3 years of treatment (RAI 13.5% [95% CI, 10.3–16.6]), decreasing the risk of getting cancer during 3 years of treatment (RAI  12.8% [95% CI, 9.9–15.7]), increasing the probability of eyebrow regrowth after 24 weeks of treatment (RAI  11.7% [95% CI, 8.8–14.5]), reducing the risk of getting blood clots during 3 years of treatment (RAI  10.7% [95% CI, 7.6–13.5]), and increasing the probability of eyelash regrowth after 24 weeks of treatment (RAI  9.2% [95% CI, 6.9–12.4]). A decreased risk of getting cancer during 3 years of treatment was the second most influential attribute for adolescents (RAI 14.1% [95% CI 10.3–17.4]), followed by increasing the probability of eyebrow regrowth after 24 weeks of treatment (RAI 13.1% [95% CI 9.4–16.3]), and increasing the probability of eyelash regrowth after 24 weeks of treatment (RAI 9.1% [95% CI, 5.6–12.2]). Relative importance of treatment‐related risks (i.e., serious infection, cancer, and blood clots) did not significantly differ for the adults (i.e., the difference between the largest and smallest RAI values was 2.79 points [*p* = 0.205]).

### 3.4 Willingness to tolerate treatment risks

The MAR results are reported in Table 3. For an increase in the probability of scalp hair regrowth from 0% to 20%, adults were on average willing to accept a mean (95% CI) 3‐year risk of serious infection, cancer, and blood clots of 7.4% (5.5–9.), 2.5% (1.9–3.1), and 9.3% (6.–12.2), respectively. For the same benefit, adolescents were willing to accept a 3.3% (2.4–4.2) increase in the risk of cancer. Risks of serious infection and blood clots were not important to adolescent patients.

**TABLE 3:** MAR of serious infections (adults), blood clots (adults), and cancer (adults and adolescents).

Attribute	Level	MAR (95% CI)			
		Adults (*N* = 201)			Adolescents^a^ (*N* = 120)
		Serious infections	Blood clots	Cancer	Cancer
Hair on most or all your scalp	1 percentage point increase	0.4 (0.3–0.5)	0.5 (0.3–0.6)	0.1 (0.1–0.2)	0.2 (0.1–0.2)
	10%	3.7 (2.7–4.6)	4.7 (3.2–6.1)	1.3 (0.9–1.6)	1.7 (1.2–2.1)
	20%	7.4 (5.5–9.3)	9.3 (6.4–12.2)	2.5 (1.9–3.1)	3.3 (2.4–4.3)
	30%	11.1 (8.2–13.9)	14.0 (9.6–18.4)	3.8 (2.8–4.7)	5.0 (3.6–6.4)
	50%	18.4 (13.7–23.3)	23.4 (16.0–30.6)	6.3 (4.7–7.8)	8.3 (6.0–10.6)
Eyebrows	1 percentage point increase	0.1 (0.1–0.2)	0.2 (0.1–0.2)	0.0 (0.0–0.1)	0.0 (0.0–0.1)
	20%	2.6 (1.6–3.5)	3.2 (2.0–4.5)	0.9 (0.5–1.2)	0.9 (0.5–1.3)
	40%	5.1 (3.2–7.0)	6.5 (4.0–9.0)	1.7 (1.1–2.4)	1.8 (1.0–2.5)
Eyelashes	0%	Reference	Reference	Reference	Reference
	20%	4.0 (2.3–5.8)	5.1 (2.7–7.5)	1.4 (0.8–1.9)	1.2 (0.6–1.8)
	40%	3.8 (2.0–5.6)	4.8 (2.5–7.1)	1.3 (0.7–1.9)	0.6 (0.0–1.2)

Abbreviations: CI, confidence interval; MAR, maximum acceptable risk.

^a^ Maximum acceptable risk of serious infection and blood clots were not computed for adolescents as changes in these two risk attributes and did not significantly influence their treatment decisions.

Adult patients were also willing to accept risks to increase the probability of hair regrowth in the eyebrows and eyelashes. For an increase in the probability of eyebrow regrowth from 0% to 20%, adults were willing to accept a mean (95% CI) 3‐year risk of serious infection, blood clots, and cancer of 2.6% (1.6–3.5), 3.2% (2.0–4.5), and 0.9% (0.5–1.2), respectively, while adolescents were willing to accept a 0.9% (0.5–1.3) increase in the risk of cancer. For an increase in the probability of eyelash regrowth from 0% to 20%, adults were willing to accept a mean (95% CI) 3‐year risk of serious infection, blood clots, and cancer of 4.0% (2.3–5.8), 5.1% (2.7–7.5), and 1.4% (0.8–1.9), respectively. For the same benefit, adolescents were willing to accept a 1.2% (0.6–1.8) increase in the risk of cancer.

## 4 DISCUSSION

This study showed that increases in treatment benefits have positive preference weights, and that patients are willing to accept non‐zero levels of serious treatment‐related risks to achieve these benefits. The most important attribute to both adults and adolescents was a 50% probability of achieving scalp hair regrowth on most or all the scalp, although adolescents cared more about scalp hair regrowth than adults. For an increase in the probability of hair regrowth, adults and adolescents were willing to accept increased risks of serious infections, blood clots, and cancer. However, the risk tolerance for serious infection and blood clots could not be estimated for adolescents. Increased risks of serious infection or blood clots were not as important to adolescents, possibly because they considered cancer as a less treatable disease and, therefore, more consequential than infections or blood clots.

A 2021 survey of 1789 US patients with AA found that the scalp was reported as the most important site of hair regrowth.^36^ In contrast to the current study, the questionnaire results suggested that few patients were willing to accept severe adverse effects to undergo treatment, that stinging or burning was the only adverse effect that most would tolerate, and that they would only accept a treatment that was “cosmetically acceptable”.^36^ However, unlike our DCE study, the 2021 survey looked at risks in isolation, did not quantify the importance of different treatment attributes, and did not elicit the trade‐offs that patients were willing to make between benefits and risks. Additionally, patients in the current study may have been more willing to accept risks because their AA was overall more severe than those in the previous survey. Whereas the current study included only patients with alopecia totalis or alopecia universalis and ≥50% scalp hair loss, the earlier survey included patients of all severities, and the extent of scalp hair loss was not included as a selection criterion.

This study has some limitations. First, patients with a clinician‐confirmed diagnosis of severe AA were recruited using different methods (e.g., physician referrals, patient organizations, and social media), and were required to obtain a completed confirmation of diagnosis, alopecia type, and extent of scalp hair loss form from their dermatologist. However, these patients were a sample drawn from an opt‐in panel of individuals who signed up to participate in healthcare research studies, which could result in self‐selection. Second, patients in this study had severe AA and the results may not apply to patients with less severe hair loss. Third, patients were recruited from the US and five European countries, and patient references may differ in other regions. Fourth, some patients may have overestimated the benefits of treatment by assuming that, if hair regrowth occurred in one part of their body, it would also occur in other parts; however, we have no evidence to suggest that this was the case. Fifth, although the adolescent sample was likely to be sufficient to estimate a main‐effects choice model, it may have been insufficient to detect statistically significant influences of the 6.0% rates of 3‐year risks of serious infections and blood clots on adolescent treatment choices. Finally, the study did not examine potential differences in preference between younger adolescents (aged 12–14 years [*n* = 61]) who completed the survey in the presence of a caregiver and older adolescents (aged 15–17 years [*n* = 59]) who completed the survey independently. Therefore, we are unable to determine whether fundamental differences in preferences exist between younger and older adolescents, nor whether the presence of a caregiver had an influence on adolescents' responses to the choice questions.

This study showed that patients with AA are willing to accept substantial risk to obtain hair regrowth, which is not surprising because AA carries a heavy socioeconomic and emotional burden.^1^, ^3^ Further, patients have limited treatment options due to a lack of targeted treatments, inconsistent efficacy, and few high‐quality randomized clinical trials demonstrating a clear benefit.^37^ This study provides important, quantitative preference information to guide the development of new treatments, inform clinical guidelines and regulatory decisions, and facilitate shared decision‐making at the point of care.^8^, ^9^, ^10^, ^11^

## FUNDING INFORMATION

This study and medical writing were paid for by Pfizer, Inc.

## CONFLICT OF INTEREST STATEMENT

Brett Hauber, Jonathan Mauer, Ernest Law, and Debanjali Mitra are employees of Pfizer, Inc. and hold stock or stock options in the company. Chiara Whichello, Myrto Trapali, Nicolas Krucien, are employees of Evidera Inc., a Thermo Fisher company, who received funding from Pfizer to conduct this study. Tommi Tervonen was an employee of Evidera Inc., a Thermo Fisher company at the time this research was conducted.

## Supporting information


Data S1.
