# Supplementary material for: Treatment preferences of adults and adolescents with alopecia areata: A discrete choice experiment
Source: J Dermatol. 2023 Dec 12;51(2):243–52. doi: 10.1111/1346-8138.17056 (PMC11483896; doi:10.1111/1346-8138.17056)
Supplement: Supplementary file 1 — Data S1. [file JDE-51--s001.docx]

# SUPPORTING INFORMATION

# Text S1. Supplemental Methods

## Targeted literature review

The targeted literature review focused on the impacts of AA on quality of life, drivers for patient perception of and treatment expectations and preferences for AA treatment outcomes and any relevant treatments, and efficacy and safety of relevant treatments. The information was used to develop candidate attributes to be explored in the qualitative patient interviews, which then subsequently informed the DCE attributes.

A search of the Ovid database (which includes key medical literature databases, such as EMBASE and MEDLINE) identified 162 qualitative (n = 49) and quantitative studies (n = 116). Among them, 145 studies were excluded through screening of the title and abstract, and a review of the full text based on the pre‑defined eligibility criteria.

Outcomes were extracted for the remaining 17 studies. The qualitative studies provided themes and subthemes, such as patient and clinician experiences and patient-reported outcomes. The extraction of quantitative studies included the preference elicitation method, plus attributes and levels.

The following were identified as attributes that may affect AA patient preferences for different treatments:

- **Benefit measures**: This included both clinical benefits and well-being benefits. For clinical benefits, reducing hair loss or increasing hair regrowth and response time were commonly reported. For well-being benefits, the psychological impact of AA and quality of life were commonly reported.
- **Risk measures**: Discrete adverse events from AA treatments were categorised into two groups by severity level—mild and severe. Mild adverse events included mild infection, gastrointestinal disorder, skin disorder, and headache. Severe adverse events included serious infection, blood disorder, cancer, and cardiovascular risks.
- **Other measures**: Mode and frequency of administration were also identified as non‑clinical measures.

## Qualitative interviews

Qualitative interviews were conducted by telephone to gain further insights into AA patients’ preferences for different treatment attributes. Two adult patients with AA in each target country (US, UK, France, Germany, Spain, and Italy; n = 12 total) were included. The overall aim of these qualitative interviews was to establish which AA impacts patients found most burdensome, understand these impacts on daily life, identify AA treatment attributes of importance to patients, and understand how much risk of AA treatment patients were willing to tolerate to achieve treatment benefits. During the interviews, patients were asked to identify any treatment attributes that were important to them.

The interviews consisted of two parts: (i) general questions to understand patients’ experiences with AA, as well as their experience with AA treatment; and (ii) specific questions to understand what aspects of AA treatment were most important to patients and to ensure that the potential AA treatment attributes were relevant to the AA patient experience.

Patients must have met all the following inclusion criteria to be eligible for inclusion in the qualitative interviews:

- Male or female patients with a dermatologist-confirmed diagnosis of AA, with ≥50% hair loss on the scalp as defined by the Severity of Alopecia Tool^36, 37^
- Aged ≥18 years
- Resident of the US, UK, France, Germany, Spain, or Italy
- Willing and able to provide electronic informed consent, indicating that they understood the purpose and procedures required for the interview and were willing to participate in the study

Patients meeting any of the following criteria were not included in the study:

- In the opinion of the screener, had a cognitive impairment, hearing problem, acute psychopathology, or insufficient knowledge of the local languages (English/French/German/Spanish/Italian) that would interfere with the ability to provide written consent and participate in the interview
- Unable or unwilling to be audio-recorded for the interview
- Other types of alopecia as diagnosed by a dermatologist
- Other diseases that affect the scalp or skin on the scalp as diagnosed by a healthcare professional
- Were experiencing hair loss due to drugs or chemotherapy.

The interviews indicated that the key AA area of hair loss was from the scalp, which was reported by all patients. Hair loss from the eyebrows and eyelashes was reported less frequently. All patients reported experiencing emotional impacts because of AA. The most frequently endorsed emotional impacts included anxiety, worry, and depression. Almost all patients indicated a lack of efficacy from the treatments they had used. Analysis of patient responses to the hypothetical scenarios showed that patients were willing to trade-off between treatment benefits and risks when making treatment decisions. An increase in scalp hair regrowth was the most important treatment attribute for patients when making treatment decisions. Non-serious side effects, such as headaches and stomach problems, were more acceptable than serious side effects.

Overall, the disease and treatment experiences reported by patients in the interviews were consistent with the findings from the targeted literature review and provided additional insights into the key treatment attributes that patients value, emphasising the perceived importance of increasing scalp hair regrowth as a key treatment benefit.

Some treatment attributes, such as the risk of headaches or mild diarrhoea, were not taken forward into the quantitative study, as they were found to be less important to patients compared to other attributes.

## Cognitive pilot interviews

The web survey was tested through a series of cognitive interviews of 15 patients with AA from six countries (US, UK, France, Germany, Italy, and Spain). Three groups of patients were recruited in separate, consecutive periods. Patients were asked to review and provide feedback on the wording, understanding, and interpretation of questions in the introductory text and DCE. The survey was updated iteratively based on the feedback collected during the three waves of interviews.

Four additional choice tasks were included in the DCE for pre-testing:

- Task 15, which compared one treatment against no treatment
- Task 16, which excluded the eyebrow and eyelash attributes
- Task 17, which tested the eyebrow and eyelash attributes presented with the levels as text qualifiers (e.g., moderate, almost complete/complete) instead of probability grids
- Task 18, which tested an alternative presentation of the scalp hair attribute with the levels as text qualifiers (e.g., hair on most of your scalp, hair on the entire scalp, no new hair on your scalp)

Patients were specifically probed on the DCE choice tasks 1, 5, 15, 16, 17, and 18 to distribute the questions throughout the exercise. Feedback on other questions was also considered for the refinement of the instrument design.

Survey updates were made based on patient feedback from the previous waves. In parallel to completing the last cognitive pilot interviews, FDA feedback necessitated the following changes:

1. Describing that the initial benefits of the treatment may not continue in the long-term
2. Describing probabilities in a similar manner (e.g., as experiencing the outcome) in the warm-up questions of both benefits and risks attributes
3. Listing the most common types of cancer associated with JAK inhibition (lymphomas and non-melanoma skin cancers)

These were reviewed by health literacy experts, who further validated the survey with one patient in December 2020. For the changes described in items #1 and #3 above, the updates were fully understood. For #2, the scalp hair attribute warm-up question was updated to include, in the description of benefit outcome possibility, that “these people may regrow some of their scalp hair (up to 80%) during treatment”—leading to the final wording presented in Table 1.

## Statistical methods

DCE data were analysed within the random utility maximisation framework.^27, 29, 30^ This framework assumes that each patient (n) chooses the alternative (j) in each DCE choice task (t) that results in the highest utility. Utility is defined as:

| 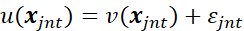 |  |
| --- | --- |

where the systematic utility component
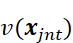

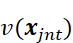
 is a linear and additive function of the treatment attributes’ levels, and
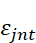

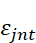
 was specified as an extreme value type 1 error, such that choice data can be analysed with logistic regression models.

The identification of the reference choice model for the analysis of patients’ preferences followed a rigorous four-step approach.

The first step was to determine whether the extra information from the follow-up choice questions between the two treatment options (i.e., the forced-choice question between options A and B when patients first preferred “No treatment”) could be used to obtain more precise estimates of preference parameters. This verification was performed by estimating separate models for the initial and follow-up choices and comparing the two sets of preference estimates. Follow-up choices were used to obtain more precise preference estimates if and only if the determinants of the initial and follow-up choices were similar. A linear regression model was estimated to evaluate the level of agreement between the two sets of estimates.

The second step was to determine whether the dummy-coded specification of the MNL model could be refined, specifically by treating some of the attributes as continuous instead of categorical variables. This simplification is appropriate when the underlying assumption of linearity in preferences is acceptable. This was verified by estimating MNL models with linear coding for each attribute separately and comparing statistical performance with the dummy-coded MNL model.

Simple scope tests were performed to investigate patients’ processing of the probability information, i.e., whether they recode quantitative attributes, such as risk levels (e.g., 0.1%, 3%, 6%) into qualitative information (e.g., low, moderate, high). Following Johnson and colleagues^38^ and Janssen and colleagues,^26^ a simple scope effect was investigated by examining changes in the difference in utility for the middle level of each risk attribute between a linear and categorical specification of the utility function. This linear estimate was then used to infer the utility of the middle risk level. The SE of this inferred utility was obtained with the Delta method.^32^ A z-test was used to determine whether the two utilities for the middle risk level significantly differed between the two specifications:

| $z-statistic=\frac{\left\vert U_{categorical}-U_{linear} \right\vert}{\sqrt{{SE}_{categorical}^{2}+{SE}_{linear}^{2}}}$ |  |
| --- | --- |

where U_categorical_ and U_linear_ correspond to the utility of the middle risk level obtained with the categorical model and linear model, respectively.

The third step was to verify that the choice data from the different countries could be combined. This was verified by estimating a heteroscedastic version of the MNL model (HMNL), thus allowing error variance (taken as a proxy measure for choice consistency) to differ across countries.^12, 39^

The last step was to verify whether the assumption of choices’ independence at the core of the MNL model was supported by the data. This was done by estimating an extended version of the MNL model, including additional individual-specific errors. These extra error terms allow for capturing of the choice data’s panel nature (i.e., multiple choices made by the same patient), and were included in the MNL model by allowing for random effects in the two alternative-specific constants. These individual errors were assumed to be distributed normally, with null mean and SD to be estimated. This extended MNL model was referred to as an error component logit (ECL) model.

Reference model specification

The final choice model for the analysis of sample average preferences was an ECL model treating all the attributes except for the probability of eyelash regrowth as linear variables. Separate ECL models were used for the adult and adolescent samples. The indirect utility function of the ECL model was specified as follows:

$V_{n} = \alpha_{1}{Treatment}_{B}+\alpha_{2}No\_treatment{+ \beta}_{1}\left[ hair\_scalp \right]{+ \beta}_{2}\left[ hair\_eyebrows \right]$

$${+ \beta}_{3}\left[ hair\_eyelashes.20\% \right]+\beta_{4}\left[ hair\_eyelashes.40\% \right]$$

$$+ \beta_{5}\left[ Infection \right]{+ \beta}_{6}\left[ cancer \right]{+ \beta}_{7}\left[ blood\_clots \right]+\omega_{n1}+\omega_{n2}$$

where α_1_ and α_2_ are two alternative-specific constants capturing (respectively) the systematic tendency to prefer the “Treatment B” over “Treatment A,” and the effect of “No treatment” compared to “Treatment A.” The β_1_ and β_2_ parameters measure the effects of a 1% increase in the probability of scalp regrowth and the probability of eyebrow regrowth, respectively. The β_3_ and β_4_ parameters capture the effects of discrete/categorical changes in the probability of eyelash regrowth, from 0% (reference level) to 20% and 40%, respectively. The β_5_, β_6_, and β_7_ parameters capture the effects of a 1% decrease in each of the three risk attributes. The ω_n1_ and ω_n2_ terms are two individual-specific errors that were assumed to be distributed, with null mean and SD (σ_ω1_; σ_ω2_) to be estimated. This model was estimated with 1,000 Halton draws to simulate the LL function. The starting values to initiate the optimisation process were obtained from the corresponding MNL model.

Based on specification tests, linear coding was used for most of the attributes, except for the probability of eyelash regrowth.

Heterogeneity in preferences

The ECL model was used to quantify the average sample preferences and assumed homogeneity in preferences (i.e., all patients share the same treatment preferences). This assumption was relaxed by investigating the systematic effect of personal characteristics on average preferences.

The systematic effect of changes in patients’ personal characteristics regarding their treatment preferences was captured by specifying additional interaction effects between the personal characteristics and the attributes’ levels to the ECL model. This extended ECL model was referred to as an interacted ECL (IECL) model and was separately estimated for the selected personal characteristics. The overall effect of the characteristics was then assessed by comparing the performance of the IECL and ECL models. The IECL model also tested for differences between the adult and adolescent ECL samples. Before entering the choice model, the personal characteristics were effect-coded, such that the interaction effects captured deviations from the average sample preferences.^40^

Outcome Measures

Relative Attribute Importance (RAI):

RAI was calculated to measure the importance of an attribute relative to all other attributes conditional on a range of levels. The difference in utilities between the most (β^max^) and least (β^min^) preferred level of an attribute k, provides an estimate of the relative importance of that attribute over the range of levels included in the DCE:

| $\mathrm{RAI}_{k}=100\times\frac{\hat{\beta}_{k}^{\max}-\hat{\beta}_{k}^{\min}}{\sum_{k\in[1;K]} \left( \hat{\beta}_{k}^{\max}-\hat{\beta}_{k}^{\min} \right)}$. |
| --- |

If the worst level also happens to be the reference category for the dummy-coding of the effects, then $\hat{\beta}_{k}^{\min}=0$ $\hat{\beta}_{k}^{\min}=0$. The Krinsky-Robb procedure in 10,000 iterations was implemented to compute the 95% CIs around the RAI scores.^31^

Maximum Acceptable Risk:

The MARs were calculated to quantify the trade-off that patients are willing to make between treatment attributes. The computation of the MAR was done for each risk attribute (i.e., risk of blood clots, risk of serious infections, and risk of cancer). This allowed preference measurement for different attributes on a common scale. MAR for improvement in attribute *k* is:

| $\mathrm{MAR}_{k}\boldsymbol{=}\frac{\frac{\partial v}{\partial x_{k}}}{\boldsymbol{-}\frac{\partial v}{\partial[RISK ATTRIBUTE]}}$ |
| --- |

The Delta method was used to obtain the 95% CIs.^32^

# Supplemental Figures and Tables

## TABLE S1. PRO measures.

| **Outcome** | **Description** |
| --- | --- |
| Hospital Anxiety and Depression Scale | The HADS is a 14-item measure to assesses anxiety (seven items) and depression levels (seven items).  Items are scored on a four-point scale (0–3), describing how often a symptom is experienced. Separate scores are estimated for anxiety and depression. Each score is then categorised into the following cases: “normal” (scores 0–7), “borderline abnormal” (scores 8–10), and “abnormal” (scores 11–21). |
|  |  |
| Patient Satisfaction with Hair Growth | The P-SAT is a 3-item measure used to assess the patient’s satisfaction with the amount, quality, and overall satisfaction with their scalp hair.  Items are scored on a seven-point Likert scale, from “very satisfied” (score=1) to “very dissatisfied” (score=4). Higher scores indicate greater dissatisfaction. |
|  |  |
| Alopecia Areata Patient Priority Outcomes | The AAPPO scale is an 11-item measure that examines hair loss, emotional symptoms, and activity limitations of AA.  The hair loss items ask the patient to describe their current amount of hair loss from the scalp, eyebrows, eyelashes, and body, using a five-point response scale ranging from “no hair loss” to “complete (do not have any hair on my [insert body area]).”  The emotional symptom items ask the patient to rate the impact of AA over the past week on a five-point scale, ranging from “never” to “always.”  The activity limitation items ask the patient to rate their experiences over the past week on a five-point scale, ranging from “not at all” to “completely”. |

Abbreviations: AA, alopecia areata; AAPPO, Alopecia Areata Patient Priority Outcomes; HADS, Hospital Anxiety and Depression Scale; PRO, patient-reported outcome; P-SAT, Patient Satisfaction with Hair Growth.

## TABLE S2. Internal validity.

| **Characteristic** | **Adult sample** | **Adolescent sample** |
| --- | --- | --- |
|  | **(N = 201)** | **(N = 120)** |
| **Choice dominance, n (%)** |  |  |
| Passed the test | 179 (89%) | 115 (96%) |
| Failed the test | 22 (11%) | 5 (4%) |
| **Expected probability of choosing the superior option, %** | 72% | 88% |
| **Choice stability, n (%)** |  |  |
| Consistent choices | 157 (78%) | 93 (78%) |
| Inconsistent choices | 44 (22%) | 27 (22%) |
| **Probability understanding, n (%)** |  |  |
| Failed all 3 tests | 0 (0%) | 0 (0%) |
| Passed 1 test out of 3 | 0 (0%) | 1 (1%) |
| Passed 2 tests out of 3 | 15 (7%) | 3 (2%) |
| Passed all 3 tests | 186 (93%) | 116 (97%) |
| **Serial non-participation, n (%)** |  |  |
| None | 183 (91%) | 117 (98%) |
| Always choose NO treatment | 16 (8%) | 1 (1%) |
| Always choose A | 1 (0%) | 1 (1%) |
| Always choose B | 1 (0%) | 1 (1%) |
| **DCE response time (min)** |  |  |
| Mean (SD) | 8.60 (9.67) | 33.77 (250.37) |
| Median (Q1-Q3) | 6 (4–10) | 8 (3–11) |
| **DCE response time (group), n (%)** |  |  |
| <2 min | 19 (9%) | 26 (22%) |
| 2–5 min | 52 (26%) | 18 (15%) |
| 5–10 min | 83 (41%) | 39 (32%) |
| 10–15 min | 26 (13%) | 23 (19%) |
| >15 min | 21 (10%) | 14 (12%) |
| **Dominant attribute preferences, n (%)** |  |  |
| Probability of scalp regrowth | 13 (6%) | 39 (32%) |
| Probability of eyebrow regrowth | 0 (0%) | 0 (0%) |
| Probability of eyelash regrowth | 0 (0%) | 0 (0%) |
| 3-year risk of serious infections | 0 (0%) | 0 (0%) |
| 3-year risk of cancer | 0 (0%) | 2 (2%) |
| 3-year risk of blood clots | 0 (0%) | 0 (0%) |
| None | 188 (94%) | 79 (66%) |

Abbreviations: DCE, discrete choice experiment.

## TABLE S3. Preferences of adult vs. adolescent patients (IECL model).

|  |  | **Sample average effect** | | **Subgroup marginal effect** | | | | |
| --- | --- | --- | --- | --- | --- | --- | --- | --- |
|  |  | **Overall** | | **Adults** | | **Adolescents** | | |
|  |  | **(N = 321)** | | **(N = 201)** | | **(N = 120)** | | |
| **Attributes** | **Levels** | **MLE** | **95% CI** | **MLE** | **95% CI** | | **MLE** | **95% CI** |
| Alternative Specific Constant | Treatment A | Reference | - | - | - | | - | - |
|  | Treatment B | -0.193*** | [-0.290–-0.097] | 0.069 | [-0.028–0.165] | | -0.069 | [-0.165–0.028] |
|  | No treatment | -1.486*** | [-2.016–-0.956] | 0.941*** | [0.458–1.424] | | -0.941*** | [-1.424–-0.458] |
| Probability of scalp hair regrowth | 1% increase | 0.053*** | [0.049–0.057] | -0.012*** | [-0.016–-0.008] | | 0.012*** | [0.008–0.016] |
| Probability of eyebrow regrowth | 1% increase | 0.016*** | [0.013–0.019] | -0.002 | [-0.005–0.002] | | 0.002 | [-0.002–0.005] |
| Probability of eyelash regrowth | 40% | 0.328*** | [0.185–0.472] | 0.097 | [-0.046–0.241] | | -0.097 | [-0.241–0.046] |
|  | 20% | 0.466*** | [0.342–0.590] | -0.015 | [-0.138–0.108] | | 0.015 | [-0.108–0.138] |
|  | 0% | Reference | - | - | - | | - | - |
| 3-year risk of serious infections | 1% decrease | 0.062*** | [0.039–0.086] | 0.050*** | [0.026–0.073] | | -0.050*** | [-0.073–-0.026] |
| 3-year risk of cancer | 1% decrease | 0.361*** | [0.291–0.431] | -0.032 | [-0.101–0.038] | | 0.032 | [-0.038–0.101] |
| 3-year risk of blood clots | 1% decrease | 0.047*** | [0.024–0.070] | 0.041*** | [0.018–0.064] | | -0.041*** | [-0.064–-0.018] |
| Error components | Component 1 | 0.160 | [-0.162–0.482] | - | - | | - | - |
|  | Component 2 | 3.179*** | [2.716–3.642] | - | - | | - | - |

Abbreviations: CI, confidence interval; IECL, interacted error component logit model; MLE, maximum likelihood estimate; SE, standard error.

Analysis information: Individuals = 321; Observations = 3852; Parameters = 20; LL = -2612.6; BIC = 5390.3; APR = 37.79%.

*** P-value <0.1%, ** P-value <1%, * P-value <5%.

## TABLE S4. RAI.

|  |  | **Adults** | |  | **Adolescents** | |
| --- | --- | --- | --- | --- | --- | --- |
|  |  | **(N = 201)** | |  | **(N = 120)** | |
| **Attribute** |  | **RAI** | **95% CI** |  | **RAI** | **95% CI** |
| Probability of scalp hair regrowth |  | 42.1 | [38.7–45.3] |  | 61.6 | [56.2–65.1] |
| Probability of eyebrow regrowth |  | 11.7 | [8.8–14.5] |  | 13.1 | [9.4–16.3] |
| Probability of eyelash regrowth |  | 9.2 | [6.9–12.4] |  | 9.1 | [5.6–12.2] |
| 3-year risk of serious infections |  | 13.5 | [10.3–16.6] |  | 1.4 | [0.1–5.4] |
| 3-year risk of cancer |  | 12.8 | [9.9–15.7] |  | 14.1 | [10.3–17.4] |
| 3-year risk of blood clots |  | 10.7 | [7.6–13.5] |  | 0.7 | [0.1–4.6] |

Abbreviations: RAI, relative attribute importance; SE, standard error; CI, confidence interval.

Model information: Individuals = 321; Observations = 3852.
